# The application of ultrasonography in the differential diagnosis of orbital lesions

**DOI:** 10.3389/fopht.2025.1679711

**Published:** 2026-08-20

**Authors:** Bernadete Ayres, Hakan Demirci

**Affiliations:** Department of Ophthalmology and Visual Sciences, University of Michigan, Ann Arbor, MI, United States

**Keywords:** orbit, imaging, ultrasound, diagnosis, tumors, inflammatory diseases

## Abstract

The imaging studies play an important role in the diagnosis and management of orbital lesions. This article provides a comprehensive overview of ultrasonographic imaging features and its practical application in the evaluation of orbital lesions. In this article, we review the features of common orbital lesions using ultrasonography, computed tomography, and magnetic resonance imaging correlation. The topics will include tumors, inflammatory diseases, congenital malformations, and orbital trauma. Each condition is reviewed in relation to its clinical presentation and imaging characteristics.

## Introduction

Ultrasonography is a noninvasive, first-line imaging method that allows for accurate detection and tissue differentiation of orbital and periorbital lesions. The dynamic nature of the exam enables the assessment of kinetic characteristics of lesions, such as their consistency and vascularity. Ultrasonography is advantageous over other imaging modalities in that it is widely available, rapidly acquired, and relatively inexpensive. Additionally, it provides reliable parameters and can be used as a primary screening when evaluating suspected orbital lesions. The main disadvantage of ultrasonography is the lack of deep penetration around the orbital apex. Moreover, it requires a high level of expertise to perform and interpret. In this review, we provide an update on the role of ultrasonography on the diagnosis and management of orbital lesions.

Ultrasound is a high-frequency acoustic wave that consists of alternating compressions and rarefactions that propagate through both fluid and solid substances. Ophthalmic ultrasonography utilizes frequency waves ranging from 8 to 50 MHz. As the transducer frequency increases, attenuation increases exponentially. The ultra-high frequency allows for incredibly detailed images by improving image resolution; however, penetration is limited to only a few millimeters. It is for this reason that probes operating at 50 MHz cannot be used to image the posterior area of the eye and orbit. Orbital ultrasonography uses a high-frequency transducer with a frequency range of 8 to 20 MHz, operating in A- and B-modes. A-mode is used to determine reflectivity and perform measurements. B-mode is performed using a 10 or 15MHz probe, resulting in an axial resolution of approximately 0.12 mm. The orbital diagnosis is based on the acoustic characteristics of tissue reflectivity and attenuation, as well as certain kinetic factors such as compressibility, mobility, and vascularity.

## Probe, sonographic techniques and protocols

**Figure 1:**
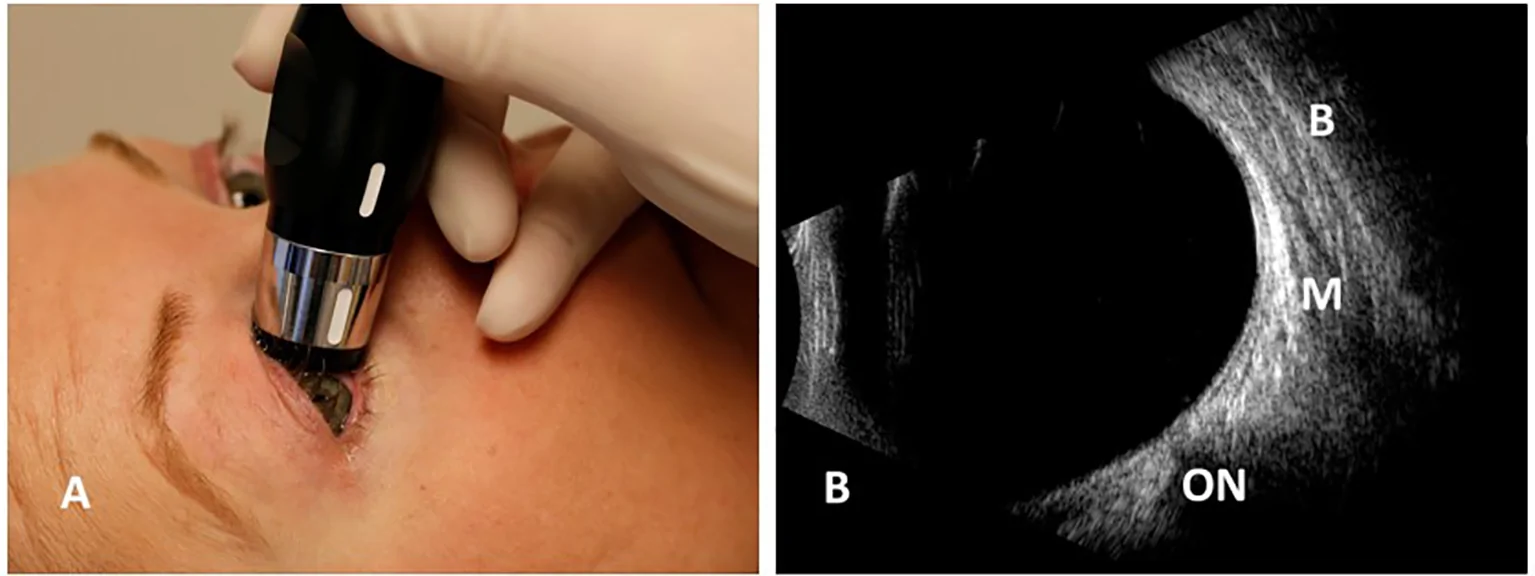
Longitudinal scan aimed temporally. **(A)** The patient is in right gaze, and the probe is placed on the nasal limbus with the marker oriented towards the cornea with the sound sweeping along a specific meridian of the temporal fundus. **(B)** The corresponding sonography of a normal eye and orbit in longitudinal view, where the optic nerve and posterior pole are imaged on the lower portion of the echogram, while the fundus periphery is imaged on the upper portion of the ultrasound. *M*, muscle; *ON*, optic nerve; *B*, lateral bone wall.

The orbital examination can be conducted in either supine or upright, with the open eye under topical anesthesia, or through the eyelids. Using the B-mode in transocular approach, the images are obtained with the probe oriented in transverse, longitudinal, and axial scans. The transverse scan produces a circumferential slice through a particular quadrant. The longitudinal scan produces a radial (or anteroposterior) slice along a particular meridian (**Figure 1A**). The axial scan with the probe is placed on the cornea of a patient in primary gaze, and the sound beam sweeps along the two opposites meridians, for example 12 and 6 on the axial vertical scan, or 3 and 9 on the axial horizontal scan.

In transverse and longitudinal scans, the probe is placed at the limbus opposite the area examined.

By convention, for transverse scan, the probe mark is oriented:

- Upright for transverse vertical scans (nasal or temporal fundus image) and for transverse oblique scans (superonasal, inferonasal, inferotemporal, and superotemporal fundus images).
- Nasally for transverse horizontal scans (superior or inferior fundus image)
- For longitudinal scans, the probe mark is oriented toward the cornea as well as the meridian that is being examined (**Figure 1A**).

The paraocular approach is used to assess the lids and anterior orbit. The probe is placed over the lids. A paraocular modified immersion technique, using a scleral shell or a finger of a glove filled with water, displays images of superficial lesions located within the eyelid or the anterior margin of an anterior orbital lesion. The orbital examination is performed methodically by screening wall quadrants and evaluating the soft tissues, extraocular muscles and optic nerve.

A Compressive history-taking, systemic and ophthalmic examinations are essential in assessing a patient with orbital pathology. Multimodality imaging with computed tomography (CT), magnetic resonance imaging (MRI), and ultrasonography (US) can provide crucial information for diagnosing and managing orbital lesions.

## Normal ultrasound characteristics of orbital structures

The orbit contains primarily fat and connective tissues, including fascia and septa, which appear as highly reflective structures encircling the globe on an ultrasound. Sound attenuation is noted in the posterior part of the ultrasound image.

On transverse or axial B-scans, the retrobulbar optic nerve appears as a hypoechoic strip, consisting of uniform nerve tissue bordered by hyperechoic meningeal sheaths.

Extraocular muscles exhibit lower echo-density compared to the surrounding fat, allowing them to be clearly distinguished. In a longitudinal scan, the full extent of these muscles can be observed, featuring a thin insertion tendon that gradually thickens into the muscle belly. (**Figure 1B**).

The bony walls of the orbit are depicted as highly echogenic, smooth, linear structures at the posterior part of the ultrasound image, followed by regions of acoustic shadowing.

The normal lacrimal gland and adjacent orbital tissue have similar acoustic impedance, making it difficult to differentiate between them under normal conditions.

## Vascular lesions

### Infantile hemangioma

Infantile hemangiomas are congenital hamartomas of blood vessels, predominately capillary size. The lesions can be superficial in the eyelid or extend deep in orbit. The tumor may be present at birth, but in most cases, the tumor becomes apparent within the first few weeks to months of life. They undergo a proliferative phase during the first year of life, followed by an involution phase over several years (1).

**Figure 2:**
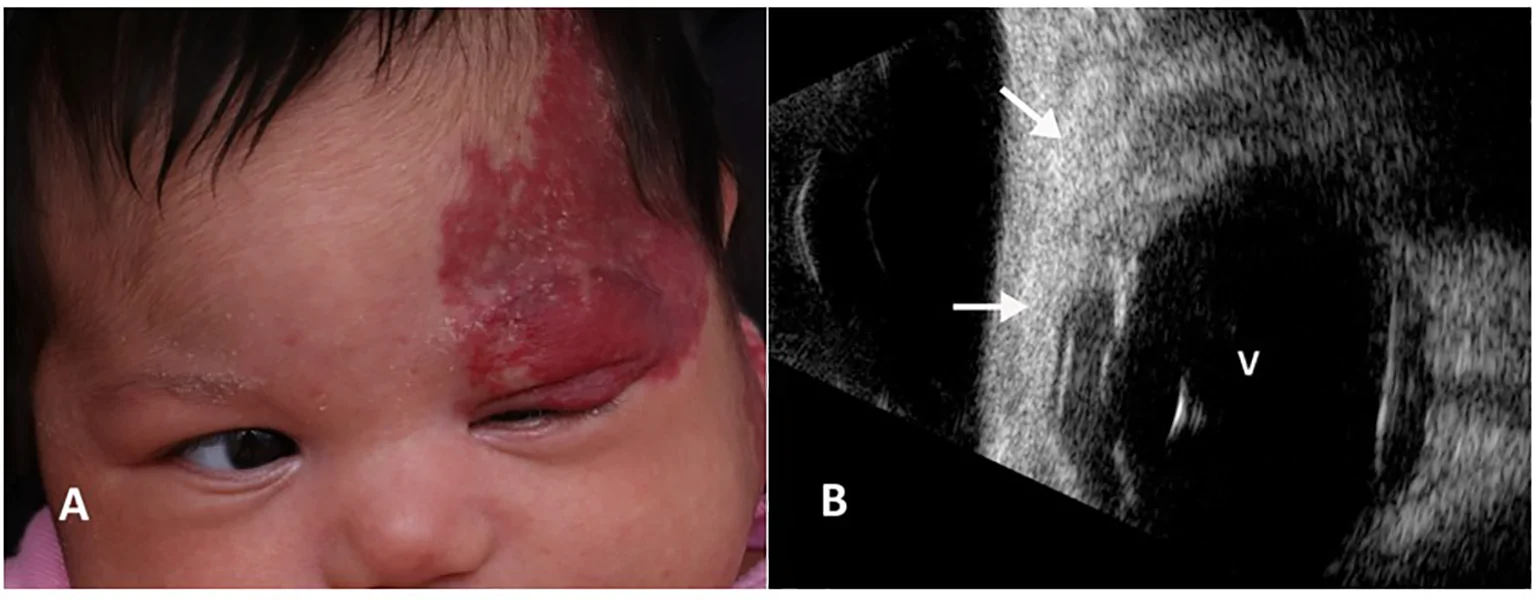
Infantile Hemangioma. **(A)** Clinical picture of a one-month-old infant shows a cutaneous hemangioma involving the left upper lid and frontal region. **(B)** Paraocular modified immersion B-can demonstrates hyperreflective lid lesion (arrows). 5*V*, vitreous cavity; *ON*, optic nerve.

On B-scan ultrasonography, infantile hemangiomas are usually poor-outlined, irregularly shaped lesions with heterogeneous internal structures, mostly high reflective. On kinetic study, the masses are compressible, and exhibit marked spontaneous internal motion consistent with vascularity (**Figure 2A, B**).

### Cavernous venous malformation

Cavernous venous malformation of the orbit, previously called cavernous hemangioma, is a common primary orbital lesion of adults. They occur more often in women, with a peak incidence in the fifth decade (2). The most common sign of cavernous venous malformation is a painless, unilateral, slow progressive proptosis from the preferential involvement of the intraconal orbital space. A significant number of cavernous venous malformations present as incidental asymptomatic lesions discovered on CT or MRI performed for unrelated reasons (3).

**Figure 3:**
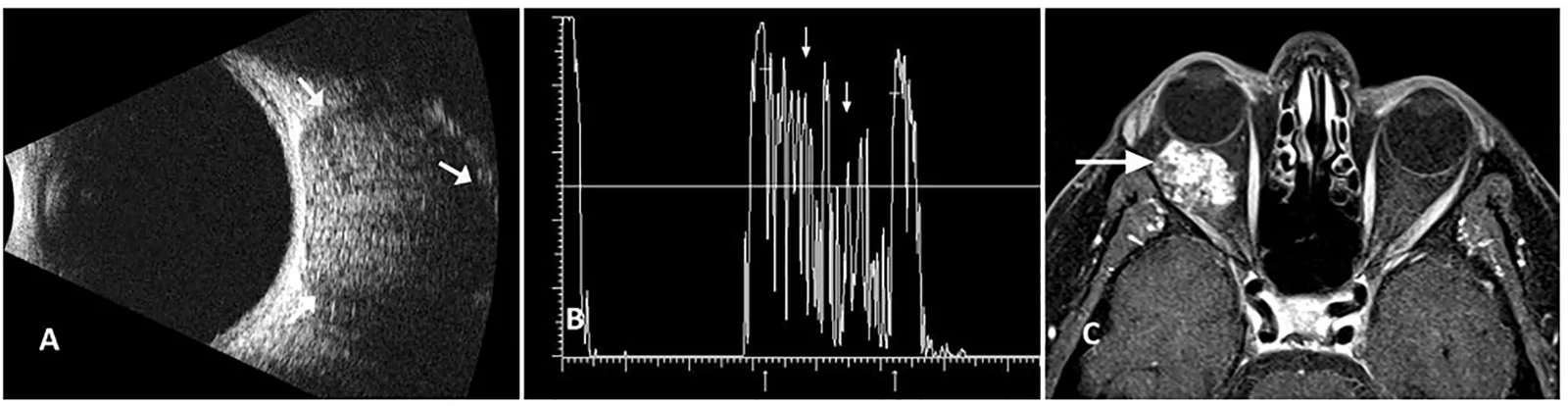
Cavernous Venous Malformation. **(A)** Transverse B-scan shows a large, well-outlined, oval shape, homogeneous orbital lesion (arrows). **(B)** A-scan shows high reflectivity and moderate sound attenuation (arrows). **(C)** Axial post-contrast T1-weighted MRI shows an intraconal mass (arrow).

On B-scan ultrasonography, cavernous venous malformation appears as a well-circumscribed round or oval lesion with a smooth surface, usually located in the intraconal space. The internal structure is regular, with medium-high to high reflectivity and moderate to strong acoustic attenuation (**Figure 3A-C**).

### Venous-lymphatic malformation

Venous-lymphatic malformation is a benign congenital tumor composed of venous and lymphatic channels. Most patients develop symptoms in the first or second decade (4).

At presentation, patients can have progressive swelling or a mass affecting the eyelids, proptosis, and restricted eye mobility. A sudden increase in proptosis indicates hemorrhage within the lesion leading to periocular pain and compressive neuropathy. Episcleral and conjunctival involvement can be present (5).

B-scan ultrasonography shows a poor-outlined, irregularly shaped lesion infiltrating the adjacent orbital tissues. The lesion is heterogeneous with low reflective lymph-filled channels separated by highly reflective septa and vessel walls.

## Tumors of hematopoietic and lymphoid tissues

### Orbital lymphoma

Ocular adnexal lymphoma can arise in the conjunctiva, eyelids, and the orbit, including the lacrimal gland. Most orbital lymphomas are of B-cell origin and affect patients older than 50 years, with a slight predominance of males (6).

These tumors usually present as an unilateral, painless masses, often causing eyelid swelling, proptosis, and palpable masses.

**Figure 4:**
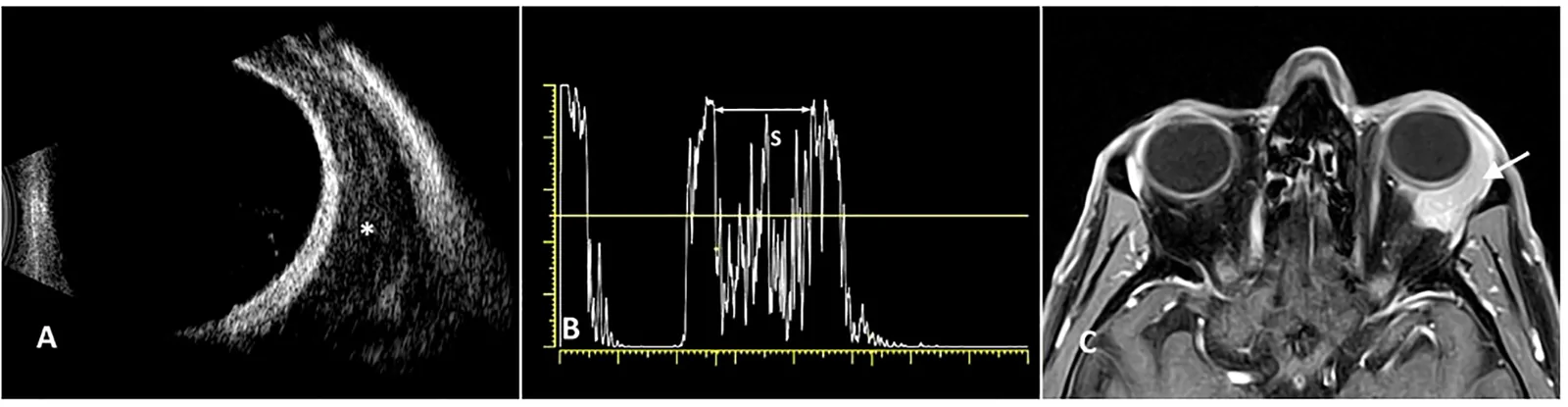
Orbital Lymphoma. **(A)** Longitudinal B-scan shows a well-outlined low-medium reflective lesion (asterisk) molding to the globe. **(B)** Corresponding A-scan displays mainly low-medium amplitude spikes (double arrow) and a distinct high amplitude spike consistent with internal septa *(S*). **(C)** MRI axial shows a large lymphoma (arrow) on the lateral region, molding around the globe.

On B-scan ultrasonography, orbital lymphoma usually presents as smooth, circumscribed, or diffuse, extraconal mass molding around the globe. The lesion shows a regular internal structure with low to medium internal reflectivity (**Figure 4A-C**).

### Orbital leukemic tumor – granulocytic sarcoma

Orbital leukemic tumors are mostly seen in the myelogenous forms, also denominated as granulocytic sarcoma, and occur most commonly in the first decade of life. These tumors may present before, concurrently, or appear during a remission stage of leukemia. The presenting features include proptosis, motility restriction, displacement of the eye, palpable mass, and pain. The involvement is usually unilateral (7). In some cases, the orbital mass preceded any hematologic manifestations at the time of presentation.

On B-scan ultrasonography orbital leukemic tumors are well-defined, homogeneous lesions with low to medium reflectivity. They are slightly compressible during kinetic examination and typically located in the extraconal space near bone remodeling areas.

### Orbital plasmacytoma

Plasmacytoma is a disease caused by the indiscriminate proliferation of monoclonal immunoglobulin-producing plasma cells and presents various clinical and pathological aspects. They are divided into multiple myeloma, solitary bone plasmacytoma, and extramedullary plasmacytoma. Orbital plasmacytoma is usually associated with multiple myeloma, diagnosed before or immediately after the initial orbital presentation (8). The median diagnosis age is 57, with equal gender distribution. Symptoms last an average of 1.9 months, and patients often present with a droopy eyelid and eye displacement on one side.

On B-scan ultrasonography, plasmacytomas are well-defined, firm lesions with regular internal structure and low internal reflectivity. Bone erosion can be detected.

## Mesenchymal tumors of the orbit

### Rhabdomyosarcoma

Orbital rhabdomyosarcoma is the most common primary malignant orbital tumor in childhood that mainly affects children in their first decade, and a higher incidence rate is noted in males.

The onset of orbital rhabdomyosarcoma can vary significantly but often ranges from days to weeks, presenting with painless proptosis with rapid swelling in an otherwise healthy child (9).

**Figure 5:**
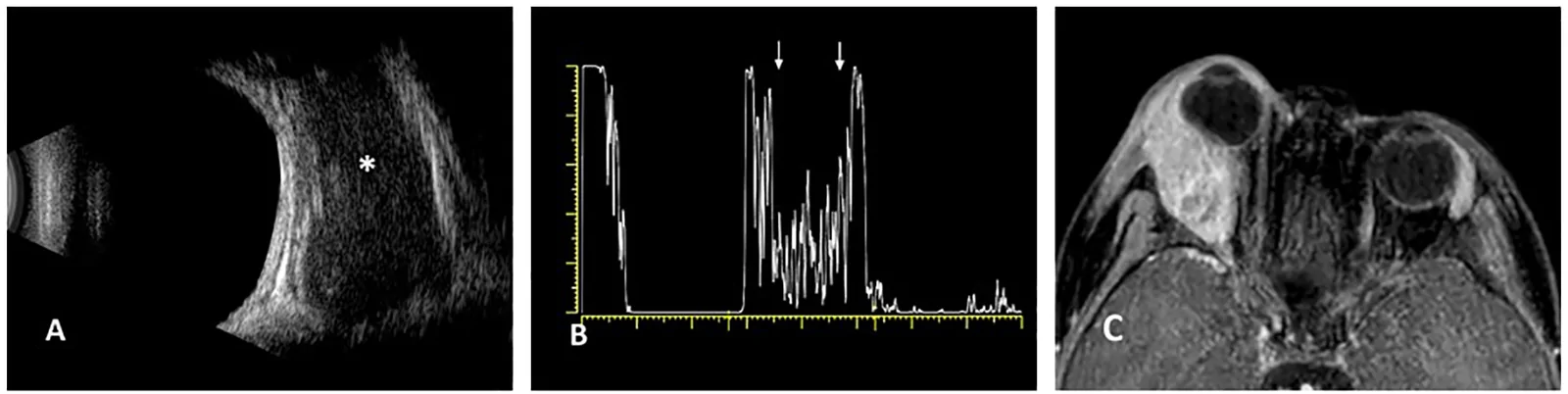
Orbital Rhabdomyosarcoma. A 9-year-old boy presented with a 4-week history of a right-sided orbital lesion. **(A)** Transverse B-scan shows a large, low reflective orbital mass (asterisk). **(B)** Corresponding A-scan shows low-amplitude internal spikes (arrows). **(C)** Axial T1-weighted MRI shows a contrast enhancement mass in the right orbit displacing the eye. The asterisk (*) is used to label an area of the figure.

On B-scan ultrasonography, orbital rhabdomyosarcoma appears as an extraconal, well-circumscribed, oval-shaped, homogeneous mass of low to medium reflectivity and can cause globe indentation. On dynamic exam the lesion is firm in consistency. Occasionally, a large lesion can invade the adjacent paranasal sinuses or intracranial contents, a finding that reflects the aggressive nature of the tumor (**Figure 5A-C**).

## Tumors of the lacrimal gland

### Epithelial tumors of the lacrimal gland

#### Pleomorphic Adenoma

Pleomorphic adenoma is the most common benign lacrimal gland tumor, representing 20% of all lacrimal gland tumors (10).

The history of symptoms lasts 1–2 years before seeing a physician. The clinical findings are a mass growing superotemporally, medial, and downward displacement of the eyeball and s-shaped ptosis.

**Figure 6:**
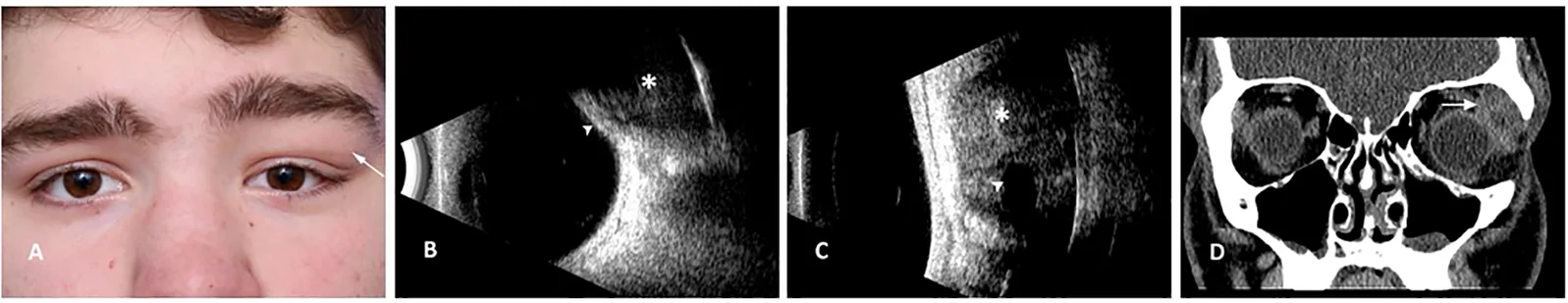
Pleomorphic Adenoma of the Lacrimal Gland. **(A)** Clinical picture of a 15-year-old male presented with a history of left upper lid swelling for one year. S-shaped left ptosis can be observed (arrow). **(B)** Longitudinal B-scan aimed superotemporally shows a large, well-circumscribed, low reflective orbital lesion (asterisk) indenting the eye (arrowhead). **(C)** Paraocular modified immersion B-scan over the lacrimal gland shows an enlargement of the gland (asterisk). **(D)** Coronal CT scan shows a left-sided orbital lesion superotemporally (arrow). The asterisk (*) is used to label an area of the figure.

On B-scan ultrasonography, pleomorphic adenoma can be either a round or oval, smooth, well-outlined mass. The internal structure is homogeneous, and the reflectivity is usually medium. The tumor is firm and can cause indentation of the adjacent eye wall and bone remodeling (**Figure 6A-D**).

#### Adenoid cystic carcinoma

Adenoid cystic carcinoma is the most common malignant epithelial tumor of the lacrimal gland. The median age for patients is 40 years. Patients typically present painful eyelid swelling, ptosis, and palpable mass superotemporally lasting approximately 6 months (10).

**Figure 7:**
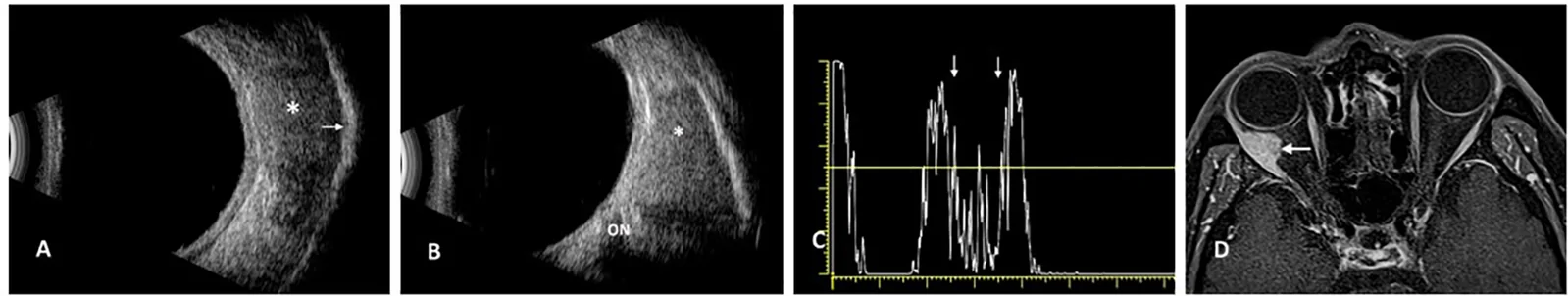
Adenoid Cystic Carcinoma of the Lacrimal Gland. A 60-year-old male presented with a history of right-sided orbital mass for two months. **(A)** Transverse B-scan aimed superotemporally shows a well-outlined large lesion (asterisk) with medium internal reflectivity. There is associated smooth bode remodeling (arrow). **(B)** Longitudinal B-scan through superior orbit shows a large mass extending posteriorly (asterisk). **(C)** A-scan displays mainly medium internal reflectivity (arrows). **(D)** Axial T1-weighted MRI shows a contrast enhanced right-sided orbital mass abutting the lateral rectus, most likely originating from the orbital segment of the gland. *ON*, optic nerve. The asterisk (*) is used to label an area of the figure.

Adenoid cystic carcinoma appears well-defined on B-scan ultrasonography in its early stages but becomes infiltrative as it progresses. The tumor shows medium to medium-high internal reflectivity and marked sound attenuation. (**Figure 7A-D**) The lesion is firm to hard, and bone erosion in advanced cases suggests malignancy.

### Lymphoid tumors

#### Lacrimal gland lymphoma

The most common lymphomas found in the lacrimal gland are non-Hodgkin type, with extranodal marginal zone B-cell lymphoma, follicular lymphoma, and diffuse large B-cell lymphoma being the most frequent subtypes. Lacrimal gland lymphomas primarily affect elderly patients with a median age in the 60s. In general, the presenting symptoms are related to the mass effect of the tumor. Thus, patients with lacrimal gland lymphomas complain of swelling, eyeball displacement, and diplopia. Pain is rarely reported. Symptom duration is on average 6 months before diagnosis (11).

**Figure 8:**
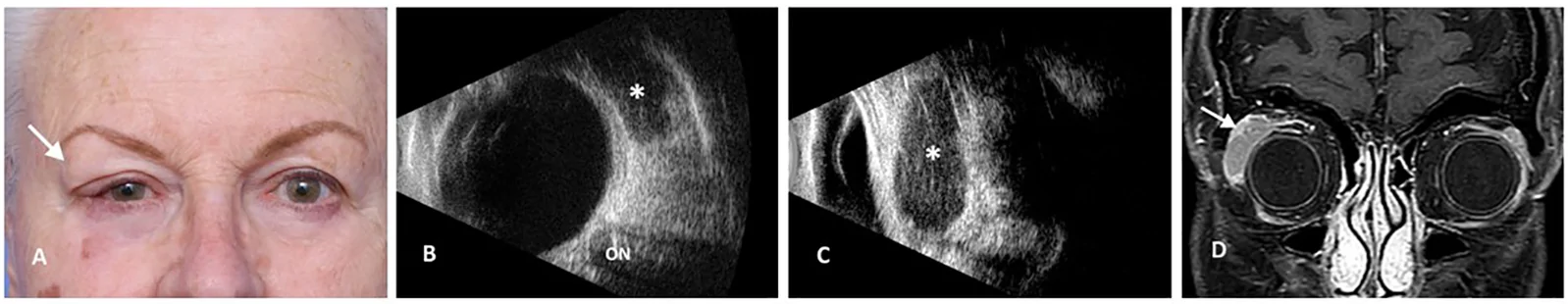
Lacrimal Gland Lymphoma. **(A)** Clinical picture of a 79-year-old female presented with right upper lid ptosis for 4 weeks. On physical exam, a palpable mass was detected superotemporally. **(B)** Longitudinal B-scan aimed superotemporally shows a large, well-circumscribed, homogeneous, low reflective orbital lesion (asterisk) **(C)** Paraocular modified immersion B-scan over the lacrimal gland shows an enlargement of the gland (asterisk). **(D)** Coronal MRI scan shows a right-sided, contrast-enhanced, oval orbital lesion superotemporally. O*N*, optic nerve. The asterisk (*) is used to label an area of the figure.

B-scan ultrasonography typically shows a well-outlined, smooth tumor with homogeneous internal structured and low to medium reflectivity (**Figure 8A-D**).

## Cysts and mucocele

A cyst is a sac-like structure lined by epithelium and containing fluid or semisolid material. These lesions are encountered across all age groups with varied clinical presentations. Orbital cysts can be classified by their relationship to adjacent structures (primary or secondary), time of onset (congenital or acquired), etiology (traumatic, surgical, inflammatory, idiopathic), or the type of cells that constitute their walls (cutaneous epithelium, conjunctival epithelium, respiratory epithelium, apocrine gland epithelium, and neural cells).

### Dermoid cysts

Dermoid cysts are congenital malformations of ectodermal origin. The lesions may contain skin appendages, including hair, bone and teeth. These can present as a palpable periocular or periorbital mass. Based on their anatomic location, they can be classified as superficial and deep. The superficial type is typically located in the extraconal region, superolateral, along the frontozygomatic suture (12). Clinically, a painless mass may be present around the eye or orbital bones; this may be soft, firm, moveable, or fixed to the deeper tissue.

**Figure 9:**
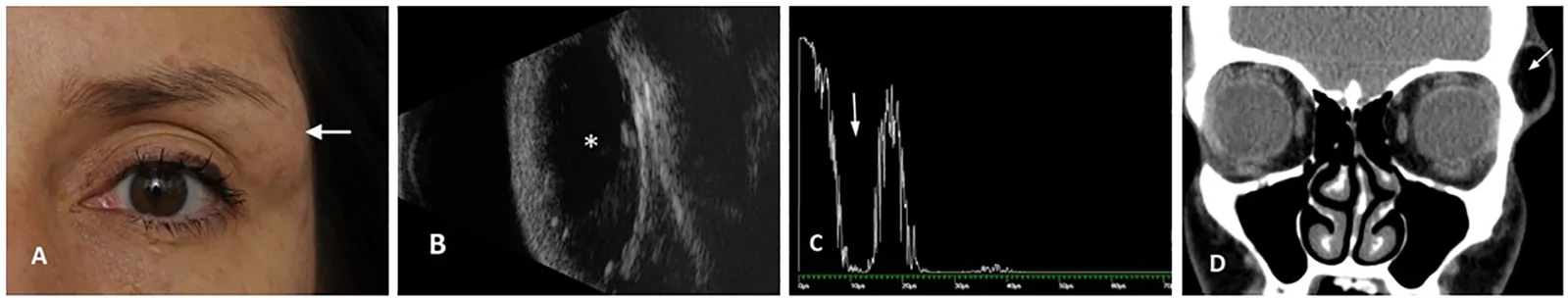
Dermoid Cyst. **(A)** Clinical picture of a 42-year-old female presented with a complaint of a large bump on the lateral left brow (arrow) that has been present since birth. **(B)** Paraocular modified immersion B-scan demonstrates a well-circumscribed, oval, homogeneous, low reflective lesion (asterisk). **(C)** A-scan displays mainly low internal reflectivity consistent with cystic lesion (arrow). **(D)** CT scan shows an oval-shaped mass centered within the anterior aspect of the right superficial temporal fossa. The asterisk (*) is used to label an area of the figure.

B-scan ultrasonography shows a well-defined, oval or rounded mass with a smooth margin. Internal reflectivity is usually low; however, cysts rich in epidermal appendages have a more heterogeneous structure and higher reflectivity (**Figure 9A-D**).

### Orbital inclusion cysts

Orbital inclusion cyst generally occurs following orbital trauma or as a late complication of ocular or orbital surgery. Giant conjunctival inclusion cysts can be a rare complication of strabismus surgery. Clinical characteristics are proptosis, eye displacement, and limitation in ocular motility.

On B-scan ultrasonography, orbital inclusion cysts are well-defined, smooth, round, or oval lesions that may be compressible. Depending on the mucoid content, their internal structure can be homogeneous with low reflectivity or heterogeneous with increased reflectivity.

### Mucocele

Mucoceles are cystic lesions that arise owing to obstruction of ostia of the paranasal sinuses, resulting in accumulation of secretions within the sinus, causing gradual expansion of the sinus with thinning of its bony walls, and eventually eroding into neighboring orbit, nasopharynx, or cranial cavity (13). Clinical characteristics are displacement of the globe, upper eyelid swelling, palpable mass, and orbital pain.

**Figure 10:**
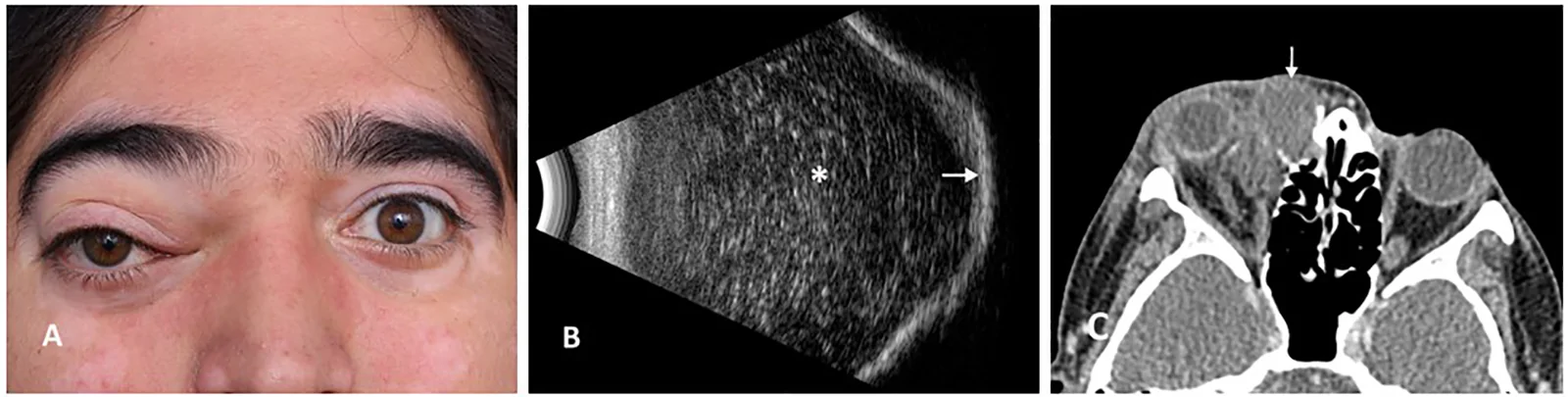
Orbital Mucocele. **(A)** External photography shows right upper lid swelling, proptosis, and inferior eye displacement in a patient presenting an orbital mass for one year. **(B)** Paraocular B-scan shows a well-circumscribed low reflective lesion (asterisk) with associated bone remodeling (arrow). **(C)** Axial CT scan shows a large expansile right fronto-orbital mucocele (arrow) and bone erosion. The asterisk (*) is used to label an area of the figure.

B-scan ultrasonography shows a large, well-outlined, extraconal, round or oval, regularly structured hypoechoic mass that extends to the adjacent sinus. The lesions usually are firm, and adjacent bone defects can be detected (**Figure 10A-C**).

### Microphthalmos with orbital cyst

Congenital microphthalmia associated with orbital cyst is an extremely rare ocular malformation. The disorder is presumed to result from incomplete closure of the embryonic orbital fissure, producing of a cyst containing cerebrospinal fluid. The clinical presentation varies from a small cyst in an apparently normal eye to a large cyst occupying most of the orbit, leaving only a small space for an eye remnant (14).

**Figure 11:**
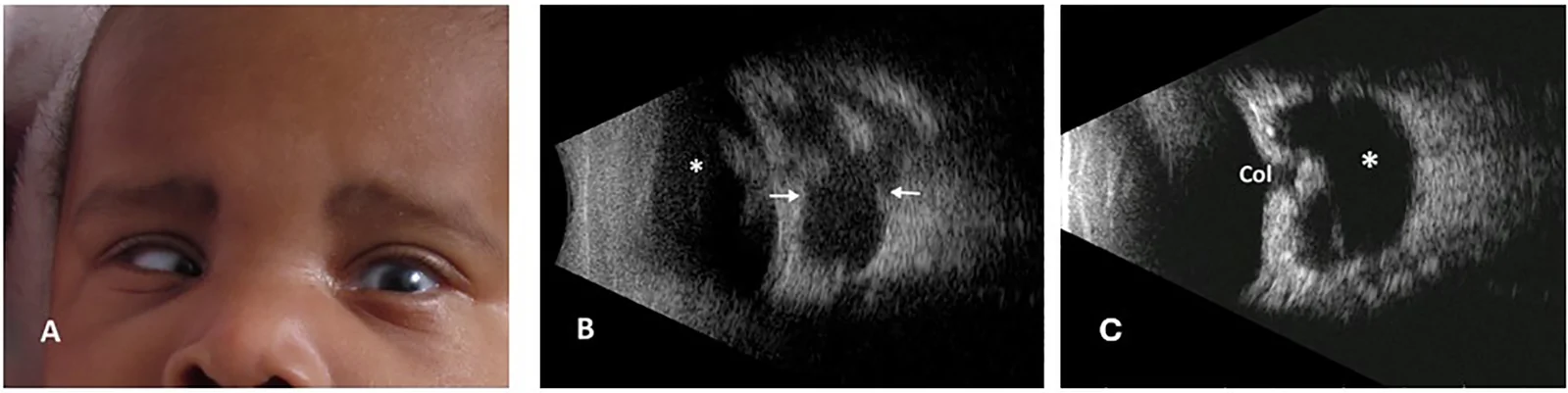
Microphthalmos with Orbital Cyst. **(A)** External photography of a 3-month-old infant with microphthalmos of the right eye. **(B)** Transverse B-scan shows a small eye (asterisk) and a large retrobulbar cyst (arrows). **(C)** Ttansvers B-scan shows a microphthalmic eye with a large optic disc coloboma and an orbital cyst. The asterisk (*) is used to label an area of the figure.

On B-scan ultrasonography, the cyst appears as a well-outlined, smooth, echolucent cavity displacing the microglobe (**Figure 11A, B**).

The combination of microphthalmos, optic nerve coloboma, and retrobulbar cyst is a very rare congenital anomaly, and most cases are unilateral and isolated. The **Figure 11C** shows a case of microphthalmos, optic nerve coloboma, and retrobulbar cyst.

## Optic nerve and peripheral nerve sheath tumors

Optic nerve tumors are classified as either those arising from the optic nerve matter or those arising from the surrounding sheath.

### Benign optic nerve glioma

Optic nerve gliomas are benign tumors categorized as pilocytic astrocytomas. The tumor is unilateral, and may occur at any age, but most become symptomatic in childhood and are also commonly seen in patients with neurofibromatosis type 1. Proptosis, visual loss, optic disc swelling or pallor, and strabismus are common clinical features at presentation (15).

**Figure 12:**
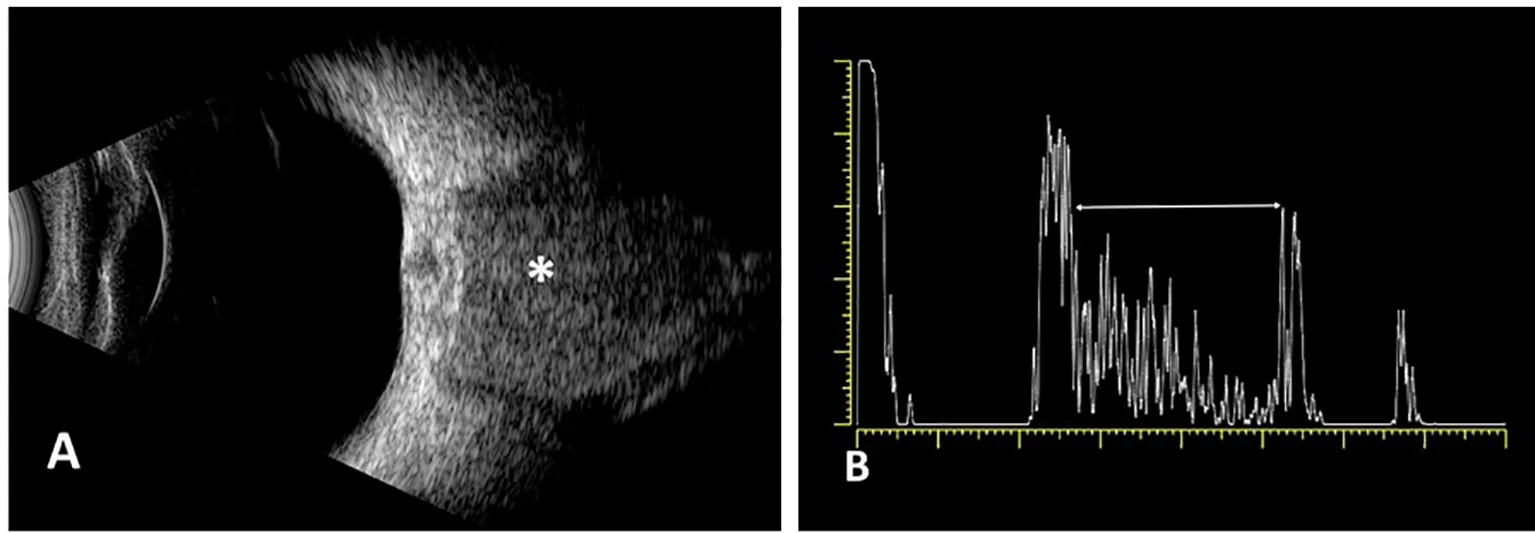
Optic Nerve Glioma. **(A)** Axial B-scan shows a large, well-circumscribed, fusiform, medium reflective enlargement of the optic nerve (asterisk), causing flattening of the globe posterior pole **(B)** Corresponding A-scan shows typical medium reflective mass (double arrow) with moderate sound attenuation. The asterisk (*) is used to label an area of the figure.

B-scan ultrasonography demonstrates a smooth fusiform or tubular enlargement of the optic nerve. The mass has a regular structure, low to medium reflectivity, and firm consistency (**Figure 12A, B**).

### Optic nerve meningioma

Meningiomas are benign neoplastic lesions arising from meningothelial cells of the meninges. Primary orbital meningiomas, originating in the optic nerve sheath, represent 1-2% of all meningiomas and are the second most common optic nerve tumor after gliomas. They primarily affect middle-aged adults. Most optic nerve meningiomas are unilateral. Patients present with gradual, progressive loss of vision. They occasionally describe transient visual obscurations as well. Proptosis and motility disturbances may also occur. Headaches are occasionally reported. Fundoscopy exams usually show optic disc swelling or disc atrophy and optociliary shunt vessels (16).

**Figure 13:**
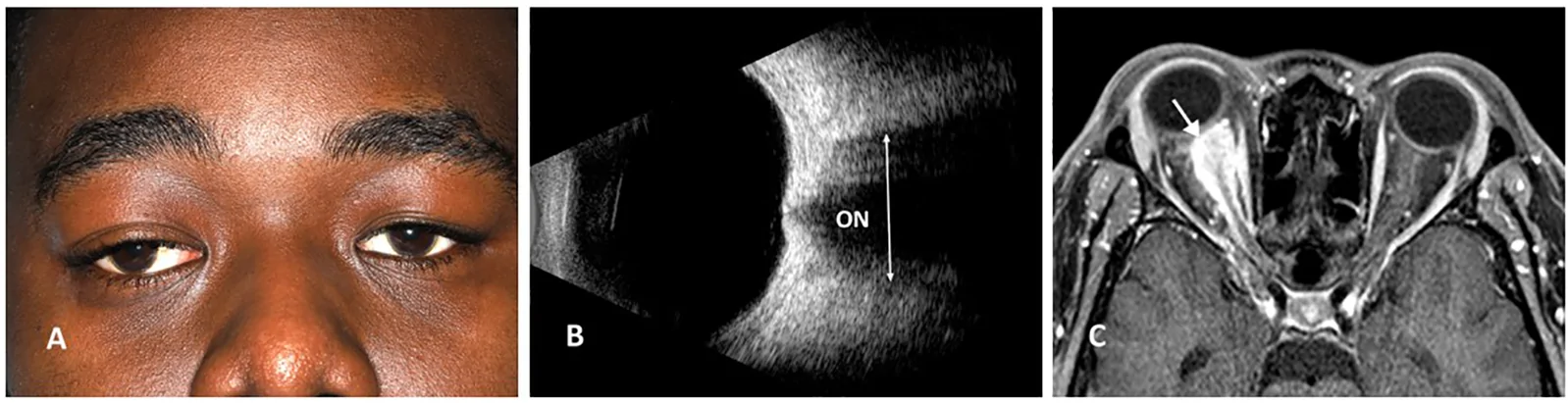
Optic Nerve Meningioma. A 24-year-old male presented with severe vision loss in the right eye for six months. **(A)** External photography shows proptosis and inferior displacement of the right eye. **(B)** Transverse B-scan shows a tubular, medium reflective enlargement of the optic nerve due to the enlargement of the meningeal sheath, with normal central neural component (double arrow). **(C)** Axial T1-weighted MRI shows an enhanced meningeal tumor surrounding the non-enhanced optic nerve matter centrally, resulting in the so-called tram-track sign (arrow). *ON*, optic nerve.

B-scan ultrasonography demonstrates a well-defined tubular widening of the optic nerve with a medium to high reflective mass surrounding the central low reflective matter of the optic nerve. Foci of calcification can occasionally be seen (**Figure 13A-C**).

### Orbital schwannoma

Schwannomas are benign peripheral nerve sheath tumors that originate from Schwann cells. Orbital schwannomas are rare, accounting for 1% to 2% of all orbital neoplasms. They are usually solitary and occur in adults in the third to sixth decade. Orbital schwannomas tend to arise from branches of the trigeminal nerve and commonly involve the superior orbit. The main symptom of orbital schwannoma is slow progressive, painless proptosis. Late manifestations include diplopia, ocular motility restriction, and mild worsening of visual acuity (17).

**Figure 14:**
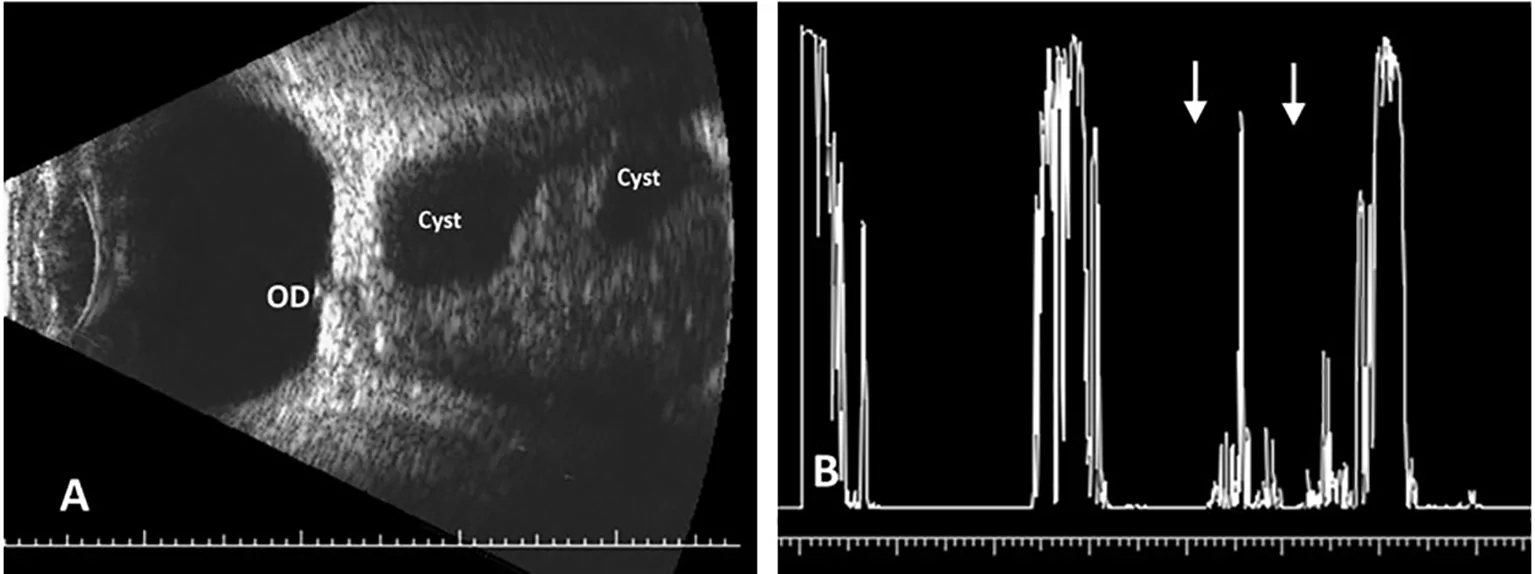
Orbital Schwannoma. **(A)** Axial B-scan shows a well-circumscribed, large retrobulbar mass abutting the optic nerve and flattening the globe posterior pole. The internal structure is irregular, secondary to multiple cysts alternating with solid, medium-reflective tumor areas. **(B)** A-scan shows extremely low intralesional areas corresponding to the cysts (arrows). *OD*, optic disc.

On B-scan ultrasonography, the lesion is typically extraconal, located at the superior orbit, and appears as a well-outlined, round, or elliptical mass with a smooth boundary. The internal structure can be variable, ranging from homogeneous low reflective pattern to heterogeneous appearance with internal cavitations. The tumor can be slightly compressible or firm. Bone excavation can be seen in ancient cases (**Figure 14A, B**).

## Orbital metastasis

The orbit is an unusual site for metastatic cancer. The incidence of orbital metastasis has increased owing to improved cancer treatment options and longer survival. Breast carcinoma and lung carcinoma are the most common primary malignancies that metastasize to the orbit. Ultrasonography provides reliable parameters and can be used as a primary screening when evaluating suspected orbital metastatic lesions. Orbital metastasis frequently manifests clinically with an abrupt onset of rapidly progressive symptoms like disturbances of ocular motility, proptosis, vision loss, and globe displacement (18, 19).

**Figure 15:**
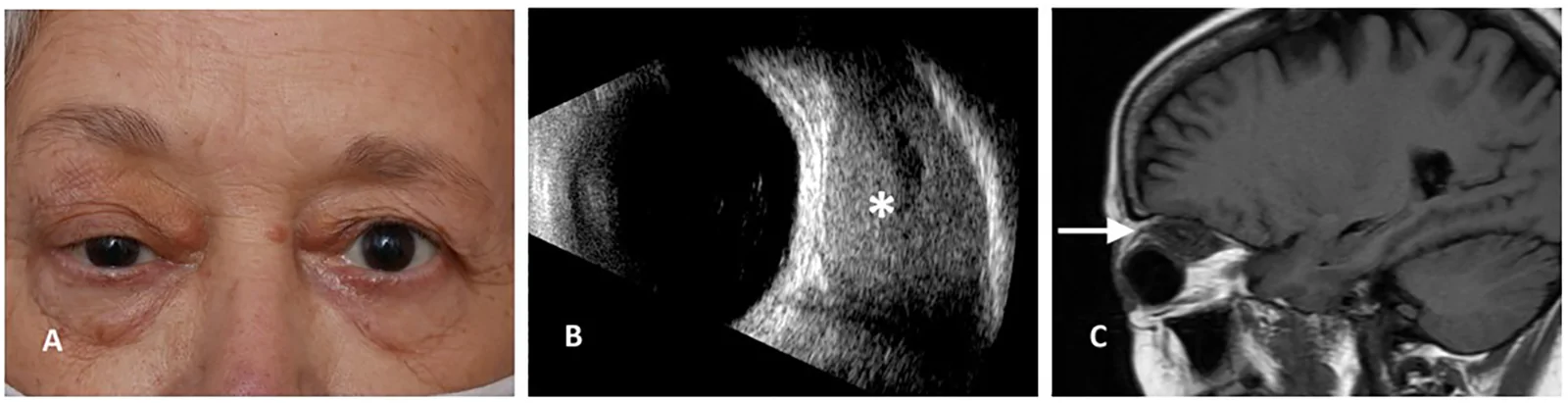
Orbital Metastasis. **(A)** External photo of a 64-year-old female presenting with proptosis, restricted eye motility, inferior displacement of the right eye, and a history of cutaneous melanoma 22 years ago. **(B)** Longitudinal B-scan aimed superiorly shows a massive, fusiform, homogeneous, low reflective enlargement of the superior rectus (asterisk). **(C)** Sagittal T1-weighted MRI shows a bulging mass of the superior rectus muscle belly, with homogenous signal intensity (arrow). The asterisk (*) is used to label an area of the figure.

On B-scan ultrasonography, most orbital metastasis demonstrates well-outlined masses with a homogeneous structure and variable reflectivity. Extraocular muscle infiltration can be found (**Figure 15A-C**).

## Orbital inflammatory disease

### Idiopathic orbital inflammation

Idiopathic Orbital Inflammation is a non-infectious inflammatory condition affecting the orbit and adnexa, with no known local or systemic cause. It can resemble lymphoproliferative diseases and thyroid ophthalmopathy, but pain is a key indicator for diagnosing this condition.

Sclerosing orbital inflammation, also known as orbital sclerosing pseudotumor, is a rare subtype of idiopathic orbital inflammation characterized by a dense fibrotic response within the orbit (20).

**Figure 16:**
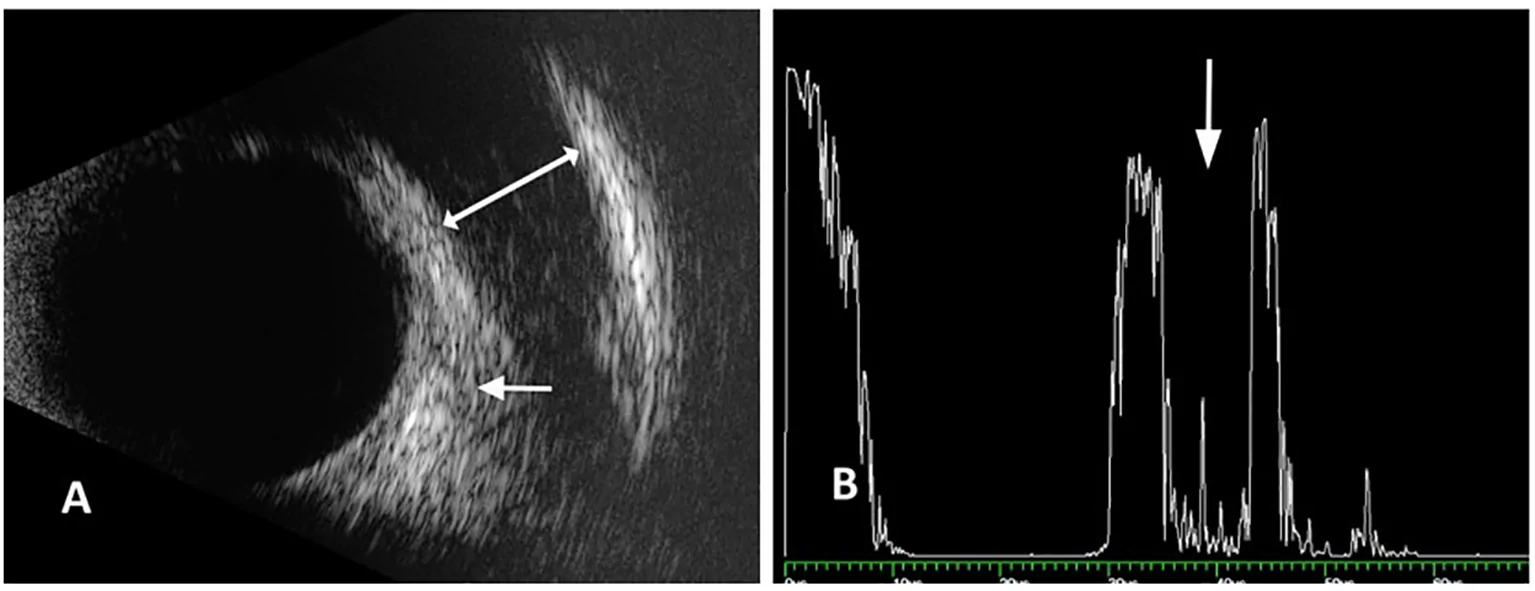
Idiopathic Orbital Inflammation – Sclerosing Subtype. **(A)** Longitudinal B-scan aimed at the superior quadrants shows a well-outlined, extraconal. regularly structured, medium reflective orbital lesion (double arrow). The lesion is outside the superior rectus (arrow). **(B)** Corresponding A-scan shows the low-reflective internal structure (arrow).

On B-scan ultrasonography, the lesion typically appears as an unilateral, multifocal, and irregularly shaped, with a regular structure and low to medium internal reflectivity. Internal vascularity may sometimes be detected. The lesion feels firm when subjected to pressure using a probe (**Figure 16A, B**).

### Idiopathic myositis

Idiopathic myositis is a specific type of idiopathic orbital inflammation. It is an acute, typically unilateral condition that causes inflammation of one or more extraocular muscles, including their tendinous insertions. Symptoms include orbital pain that worsens with eye movement, double vision (diplopia), and often some eyelid swelling, drooping of the eyelid (ptosis), and conjunctival chemosis. There may also be redness over the insertion of the affected muscle.

**Figure 17:**
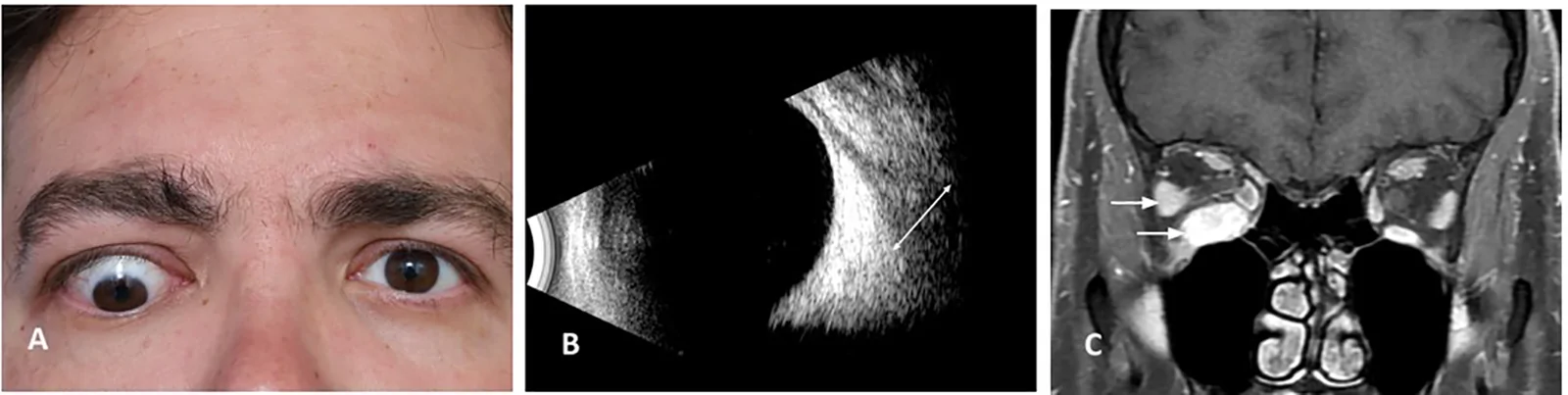
Idiopathic Orbital Myositis. **(A)** External photography of a 36-year-old patient with recurrent inflammation of the right inferior rectus. **(B)** Longitudinal B-scan shows a low reflective enlargement of the right inferior rectus (double arrow). **(C)** Coronal T1-weighted MRI shows an enlargement of the lateral and inferior recti muscles (arrows).

On B-scan ultrasonograhy exam, the affected muscle is diffusely thickened with involvement of the insertion and belly regions. The internal reflectivity is typically low (**Figure 17A-C**).

### IgG4-related dacryoadenitis

IgG4-related disease is a multi-organ fibro-inflammatory condition characterized by distinctive histopathology, including lymphoplasmacytic infiltration, an increased number of IgG4+ plasma cells, and elevated IgG4/IgG ratios (greater than 40%). The lacrimal gland is the ocular site most frequently affected (21, 22).

**Figure 18:**
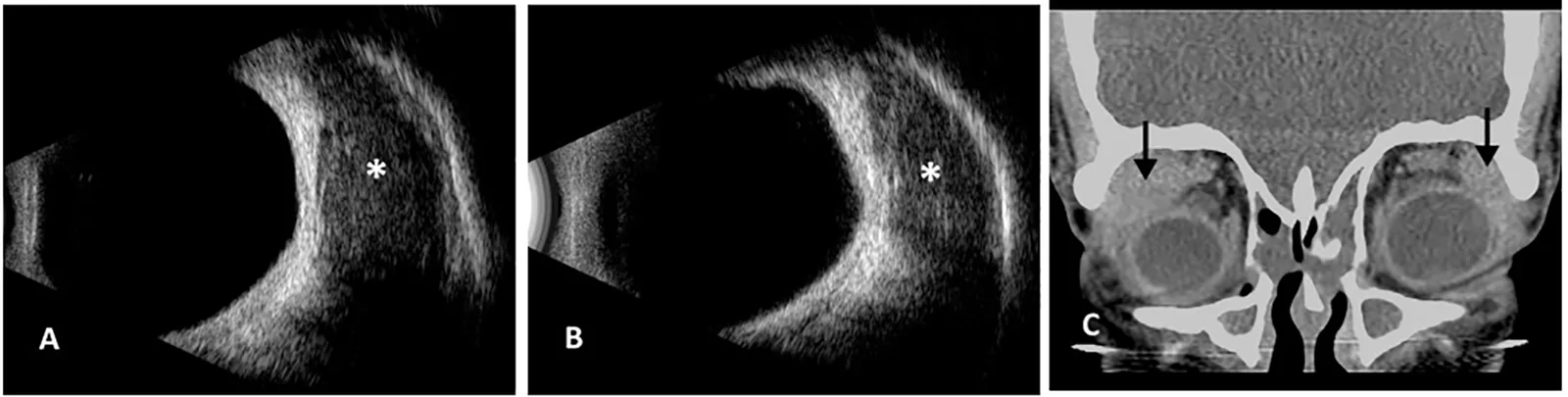
IgG4-Related Dacryoadenitis. **(A, B)** Longitudinal B-scan of the right eye and left eye, respectively, aimed superotemporally, show clearly outlined, oval-shaped enlargement of the lacrimal gland (asterisk) displaying a medium reflective honeycomb appearance. During the exam, the mass demonstrated slight compressibility when pressure was applied with the probe. **(C)** Coronal CT scan demonstrates enlarged lacrimal glands at the superotemporal aspect of the orbits (arrows). The asterisk (*) is used to label an area of the figure.

B-scan ultrasonography can reveal a well-defined, oval enlargement of the lacrimal gland that exhibits a medium reflective honeycomb pattern. The mass shows slight compressibility under the probe pressure (**Figure 18A-C**).

### Sarcoidosis

Ocular involvement in sarcoidosis is common, with anterior uveitis the most frequent manifestation. Orbital and adnexal involvement can occur in 8 to 10% of patients (23, 24). Within the orbit, sarcoidosis can affect the lacrimal gland, soft tissue, extraocular muscles, and optic nerve. The lacrimal gland is the most common site affected by extraocular sarcoidosis. The primary signs and symptoms at the initial presentation of orbital and adnexal sarcoidosis include eyelid swelling, a painless palpable eyelid mass, eye protrusion (proptosis), drooping eyelid (ptosis), discomfort, limited eye movement, dry eyes, double vision (diplopia), and reduced vision. Patients may have been previously diagnosed with systemic sarcoidosis, or the orbital and adnexal involvement may be the first sign of the disease. Chest X-ray usually reveals hilar lymphadenopathy in cases of systemic sarcoidosis. Definitive diagnosis relies on histopathologic analysis with the presence of characteristic non-caseating granuloma.

**Figure 19:**
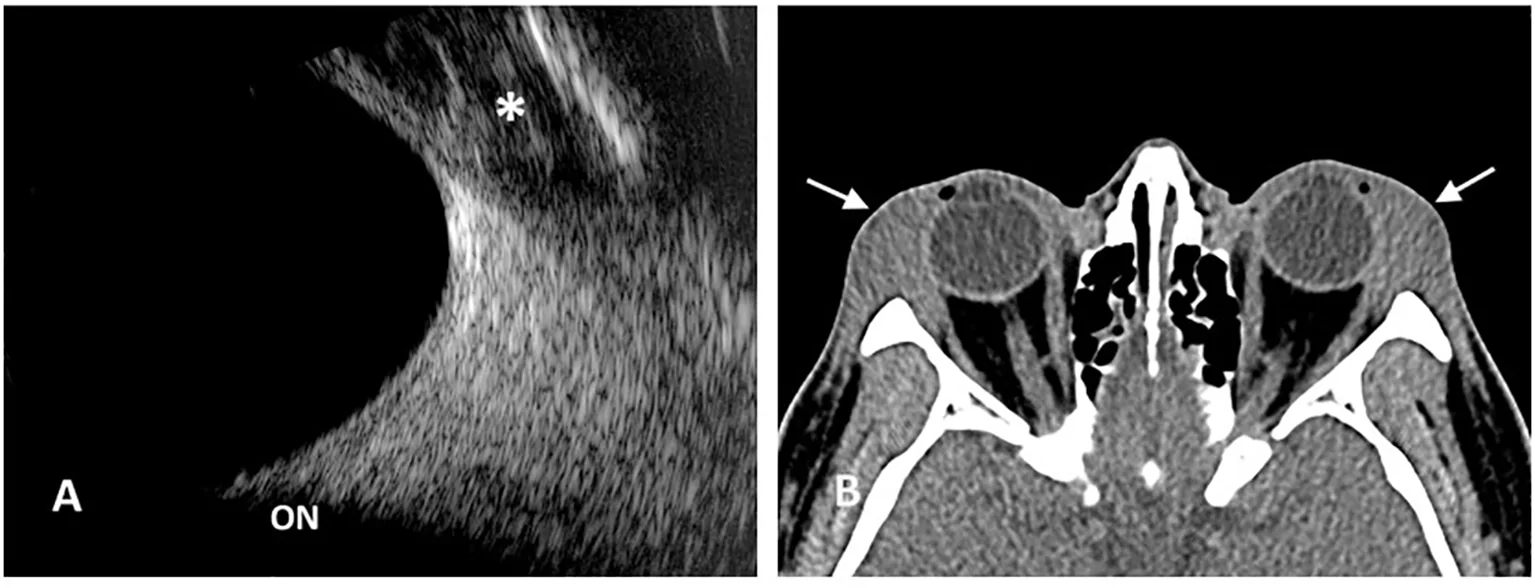
Sarcoid Dacryoadenitis. **(A)** Longitudinal B-scan right eye aimed superotemporally shows a well-defined, homogeneous, medium reflective enlargement of the gland (asterisk). **(B)** Axial CT scan shows bilateral lacrimal gland enlargement (arrows). *ON*, optic nerve. The asterisk (*) is used to label an area of the figure.

On B-scan ultrasonography, patients with sarcoid dacryoadenitis show well-outlined, somewhat homogeneous, low to medium reflective enlargement (**Figure 19A, B**).

### Thyroid eye disease

Thyroid eye disease is an autoimmune condition closely linked to Graves’ disease. Common clinical signs include redness and swelling of both eyelids, chemosis, exophthalmos, retraction of the upper eyelid, and limited eye movement. This disease typically affects the belly of the extraocular muscles while sparing the tendinous insertions. In a small number of patients, orbital involvement can appear before any endocrine abnormalities (25).

**Figure 20:**
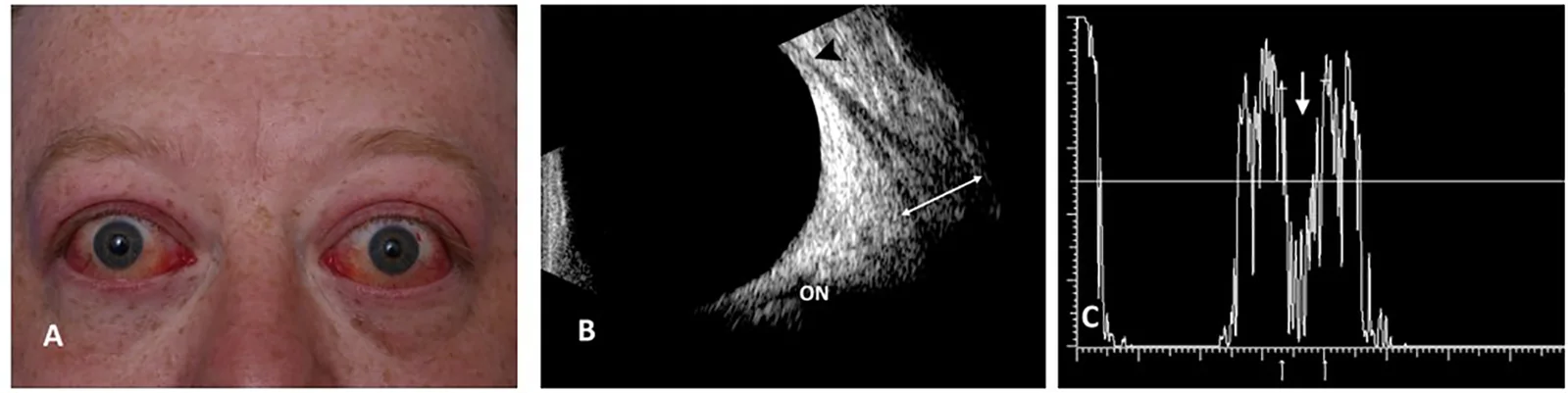
Thyroid Eye Disease. **(A)** External photography shows bilateral proptosis, upper and lower lid retraction, and injected conjunctiva. **(B)** Longitudinal B-scan of the right inferior rectus shows an enlarged muscle belly (double arrow) and normal muscle tendon (arrowhead). **(C)** Corresponding A-scan ultrasound of the right inferior muscle demonstrates low internal reflectivity. (arrow). *ON*, optic nerve.

B-scan ultrasonography demonstrates bilateral, symmetrical enlargement of multiple muscles. Typically, the muscle bellies are expanded while the tendons remain unaffected (**Figure 20 A** - C). During the active inflammatory phase, the internal reflectivity of the enlarged muscles may be low, increasing the reflectivity in the fibrotic phase. Additional manifestations include swelling of the orbital fat, low-reflective thickening of the periorbita, and enlargement of the lacrimal gland.

## Orbital trauma

Orbital injuries are often seen in patients with multiple traumas. Blunt, penetrating, or perforating injury to the eye and orbit can result in hematoma, inflammation, or retained foreign body.

### Orbital foreign body – pellet gun

A careful history and physical exam may point to suspicions about an orbital foreign body.

B-scan ultrasonography is a valuable addition to CT scans to detect and reliably localize orbital foreign bodies. Pellet guns and other spherical foreign bodies produce long chains of rapidly successive echoes which gradually decrease intensity – comet tail artifact.

## Summary

The orbit contains a wide variety of tissues and structures that can give rise to different pathological conditions in both adults and children. Ultrasonography is a readily available, first-line imaging technique for detecting and differentiating orbital lesions. By understanding the clinical presentation and ultrasound characteristics of these conditions, healthcare providers can narrow down the differential diagnosis and arrive at a final diagnosis. This information is crucial for the management, treatment, and monitoring of orbital pathological processes.
